# DEENet: an edge-enhanced CNN–Transformer dual-encoder model for steel surface defect detection

**DOI:** 10.1038/s41598-026-36390-9

**Published:** 2026-01-30

**Authors:** Weihua Pan, Ruijie Zhong, Junchuan Huang, Ye Li, Wenyuan Zhang, Ting Liu, Yujie Liu

**Affiliations:** 1 Guangzhou Institute of Science and Technology, Guang Zhou, 510540 China; 2 School of Computer Sciences, Universiti Sains Malaysia, 11800 Gelugor, Pulau Pinang Malaysia

**Keywords:** DEENet, Dual-encoder, CNN–transformer backbone, Edge-enhancement, Industrial inspection, Engineering, Mathematics and computing

## Abstract

Steel is an indispensable material in modern industry, and its surface quality directly affects the performance and service life of products. To address problems of insufficient feature extraction capability, weak detection of small defects, and blurred target contours that lead to degraded edge information in steel surface defect detection, this paper proposes a novel edge-enhanced dual-branch steel surface defect recognition model, DEENet. First, a dual-encoder module based on CNN and Transformer is designed to extract image features and enhance the feature extraction capacity of the backbone network. Second, a Dual Channel Fusion module is introduced to perform cross-enhancement between the local features captured by the CNN and the global semantic features modeled by the Transformer, achieving feature complementarity and improving the detection accuracy for small defects. Finally, an edge enhancement module, C2f_EEM, is designed to highlight gradient differences between defective and normal regions through differential operations, thereby strengthening contour information and improving the model’s sensitivity to defect edges. Experimental results on the NEU-DET dataset show that, compared with other algorithms, DEENet achieves a superior mean Average Precision (mAP) of 81.4%, enabling more accurate detection of steel surface defects and providing valuable reference for defect inspection in real-world production scenarios.

## Introduction

**Fig. 1:**
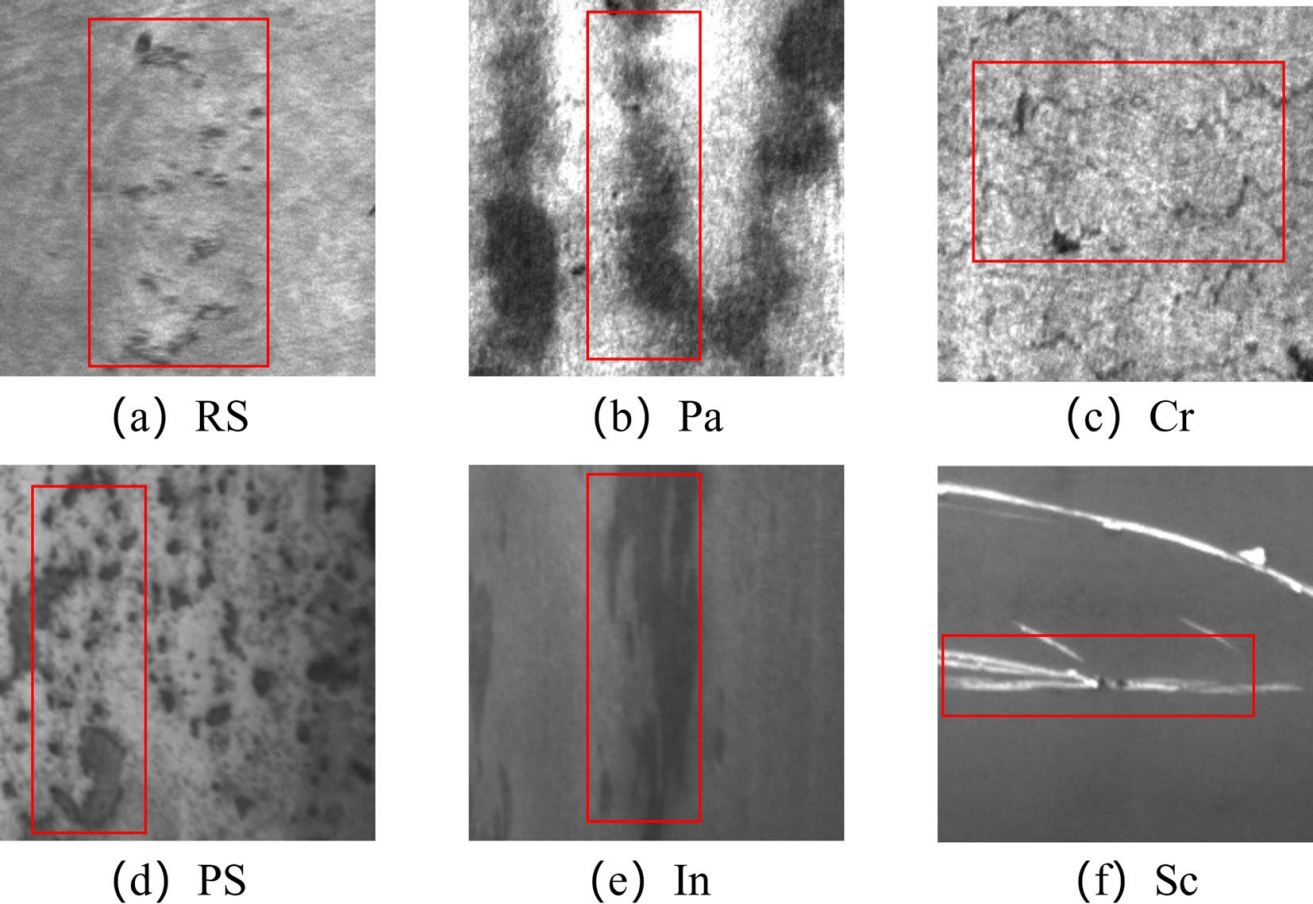
Common types of defects on the surface of steel strip.

Steel is an indispensable foundational material in modern industry and has a major impact on the manufacture and safety of a wide range of industrial products. During steel production, surface defects can be introduced by fluctuations in process conditions, equipment wear, and variations in raw-material quality^1^. As illustrated in Fig. 1, typical defect categories include rolled-in scale (RS), patches (Pa), crazing (Cr), pitted surface (PS), inclusion (In), and scratches (Sc). These defects not only degrade the performance and service life of steel but also pose serious safety hazards for subsequent processing and use^2,3^. Consequently, surface-defect inspection is critical for ensuring product quality in steel manufacturing^4^. With rapid advances in computer vision and deep learning, image-based methods for steel surface defect detection have been extensively studied and applied^5^. Traditional approaches^5–7^ rely primarily on manual inspection or simple image-processing techniques, which suffer from slow throughput, limited accuracy, and substantial human bias. By contrast, deep learning object detection algorithms can automatically learn and extract discriminative features from images, enabling efficient and accurate detection of steel surface defects.

Among object-detection algorithms, the YOLO (You Only Look Once) family^8–10^ has garnered wide attention for its efficiency and accuracy. By predicting the locations and categories of all objects in a single forward pass, YOLO achieves end-to-end detection. However, because defect morphology (shape, size, texture) varies widely and industrial backgrounds are complex and highly variable, the original YOLO variants^11–13^ struggle to robustly detect tiny and low-contrast defects and to maintain precise localization under cluttered backgrounds in steel surface–defect detection. It is therefore necessary to tailor YOLO-based methods with stronger multi-scale feature extraction, small-object sensitivity, and edge-aware representations to the specific requirements of this task. Lu et al.^14^ proposed an improved YOLOv5s-based model that incorporates the ULSAM attention mechanism to enhance contextual feature fusion and the extraction of small targets, but the resulting detection accuracy remained relatively low, especially for small and densely distributed defects. Lv et al.^15^ developed an enhanced YOLOv7 by adopting the lightweight CARAFE upsampling operator, an integrated cascade attention mechanism, and a decoupled head, effectively improving overall accuracy and speed; however, its performance on challenging defect categories and complex backgrounds still fell short of industrial requirements. Building on YOLOv8s, Zhang et al.^16^ introduced a C2f-Triplet module and the CARAFE upsampling operator to boost accuracy; nevertheless, performance on small targets remained suboptimal. Zhang et al.^17^ further increased the model’s attention to small-defect features through multi-scale parallel processing and fusion of shallow and deep features, yet their approach still exhibited weakened edge representations and missed detections under severe background interference. Overall, these YOLO-based improvements partially alleviate the limitations of the original series but do not fully resolve the combined challenges of multi-scale feature extraction, robust detection of small defects, and edge-preserving localization in real industrial scenarios.

Despite advances in both traditional and contemporary techniques for steel surface defect detection, persistent bottlenecks remain, as existing methods still struggle to learn discriminative features under complex backgrounds, to reliably detect tiny or densely distributed defects, and to accurately localize local structures and defect edges, which in turn limits both detection accuracy and inspection efficiency in real industrial settings. A deeper analysis reveals three key issues: (1) Traditional methods exhibit limitations in feature extraction. They struggle to simultaneously capture fine-grained local details and broader global context, so detection performance degrades when confronted with defects of diverse shapes and scales. (2) Many existing models perform inadequately on small-object detection. They have difficulty reliably distinguishing defects that are very small or densely distributed, which constrains both overall accuracy and robustness. (3) Current approaches demonstrate weak sensitivity to edge information along object contours. The boundaries between defective and normal regions are often indistinct, leading to attenuated edge features and ultimately hindering precise defect identification.

To overcome the aforementioned limitations, we propose DEENet, a novel detector for steel surface defects. Extensive experiments demonstrate that DEENet substantially enhances feature representation under complex backgrounds, improves detection of small and densely distributed defects, and strengthens edge localization, thereby increasing detection accuracy while maintaining low computational cost, providing a viable basis for future deployment in industrial production lines.

The primary contributions of this study are as follows:

1. We design a backbone integrating a convolutional encoder and a Transformer encoder to strengthen feature extraction. The dual-path structure captures local details and global context, improving defect representation across different shapes, scales and textures.
2. We introduce a Dual-Channel Fusion module that cross-enhances local CNN features with global Transformer semantics. This fusion improves the precision and robustness of small-defect detection, especially in dense or cluttered scenes.
3. We develop an edge-enhancement module, C2f_EEM, which fuses boundary cues with deep semantics to mitigate contour-detail loss. By strengthening edge features and clarifying defect boundaries, it reduces misclassification near edges.

The remainder of this paper is organized as follows. Section “Related works” reviews related work on traditional steel surface inspection methods and deep learning–based object detectors. Section “The overview of methods” presents the proposed DEENet detector in detail, including its dual-encoder backbone, Dual-Channel Fusion module, and edge-enhancement module C2f_EEM. Section “Experiments” describes the experimental setup, datasets, evaluation metrics, and reports extensive comparative and ablation studies that validate the effectiveness of DEENet. Section “Discussion” and Section “Conclusion” summarizes the main findings and discusses potential directions for future industrial applications.

To maintain consistency in abbreviations and symbols, we have compiled all the major technical abbreviations and mathematical symbols in Table 1, and briefly explained their roles in the DEENet architecture.

**Table 1:** Abbreviations and symbols.

	Symbol	Explain
Model	DEENet	This paper proposes an edge enhancement CNN–Transformer dual encoder model
	AKConv	Adaptive-Kernel Convolution
	DCF	Dual Channel Fusion
	C2f_EEM	Edge-Enhancement Module
	CBAM	Convolutional Block Attention Module
	ViT	Vision Transformer
Evaluation indicators	mAP	mean Average Precision
	F1-score	Harmonic mean of precision and recall
	FPS	Frames Per Second
	GFLOPs	Measuring the computational complexity and resource consumption of the model
Mathematical symbols	$C_{i}$ $T_{i}$	These represent feature maps from the CNN branch and the Transformer branch, respectively
	$Q,K,V$	Query, Key, and Value in Attention Mechanisms
	$\sigma$	Sigmoid activation function
	$$F_{a}$$$$F_{b}$$	These represent the shallow and deep features in the edge enhancement module, respectively
	$\varphi (Z)$	High-frequency feature maps are used to extract edge information through differential operations
	$\oplus$	Element addition in residual join

## Related works

### Two-stage methods

In recent years, because traditional strip steel surface defect detection methods suffer from poor generalization ability, low production efficiency, and unsatisfactory product quality, deep learning–based surface defect recognition for strip steel has gradually become a research hotspot. Among deep learning approaches for defect detection, object detection algorithms have attracted wide attention, and researchers have been committed to developing more accurate and efficient algorithms to improve model accuracy and generalization. Deep learning–based object detection algorithms can be mainly divided into two categories: two-stage methods and single-stage methods. Common two-stage methods include Faster R-CNN^18^, R-FCN^19^, and Mask R-CNN^20^. For example, Xia et al.^21^ proposed four improvements based on the Faster R-CNN algorithm: a bilateral filtering algorithm, a feature pyramid network built on ResNet-50, an ROI Align operation, and the K-means algorithm, which were applied to plate surface defect detection. Weng et al.^22^ improved the Mask R-CNN algorithm used for strip steel surface defect detection by introducing the K-means II clustering algorithm to enhance the anchor generation of the region proposal network (RPN), and by removing the mask branch to adjust the network structure of Mask R-CNN, thereby improving detection accuracy and speed. Although two-stage methods perform well in terms of detection accuracy and generalization ability, their high computational complexity and considerable hardware requirements create challenges. Overall, the limited real-time suitability and high deployment cost of existing two-stage methods provide an important motivation for the development of single-stage methods and also for the design of our model.

### Single-stage and self-supervised methods

To address these problems, single-stage methods have been proposed. Common single-stage methods include SSD^23^ and the YOLO series. For example, Liu et al.^24^ improved the SSD model by integrating residual networks, feature fusion, and channel attention mechanisms, and formed the RAF-SSD network to increase detection accuracy. Wang et al.^25^ designed a strip steel surface defect detection method based on YOLOv5, which combines multi-scale detection blocks and a spatial attention mechanism. In addition, Song et al.^26^ introduced deformable convolutions, BiFPN, and attention mechanisms based on the YOLOv8 algorithm, which significantly enhanced the ability of the model to detect small targets and provided an efficient and accurate defect detection method for the steel industry. Zhou et al.^27^ integrated large-kernel depthwise convolutions and a coordinate attention mechanism, improving the sensitivity of the model to defect locations and achieving further separation between defects and background. He et al.^28^ proposed an adaptive fine-grained channel attention mechanism to reduce the number of model parameters and introduced a normalized Wasserstein distance loss to optimize the localization of small defects. Ayon et al.^29^ introduced a learnable memory module to enhance the ability of Vision Transformers to capture long-range dependencies and improve the detection accuracy of subtle defects, but the training time is long, a large amount of labeled data is required, and interpretability is limited. Xu et al.^30^ used the SimSiam self-supervised framework to pre-train on unlabeled data and then transferred the learned representations to Faster R-CNN for defect detection. Although this approach reduces the dependence on labeled data and improves scalability and generalization, the computational requirements remain high and the accuracy for some defect categories is still low. Overall, these single-stage, attention-based and self-supervised methods improve detection accuracy and reduce label dependence but still struggle with multi-scale features in complex backgrounds, robustness to tiny defects and high computation, which motivates the DEENet model in this paper to enhance feature extraction and fusion for small targets and edges within a single-stage framework.

In summary, existing steel surface defect detectors face three main bottlenecks, which DEENet aims to overcome: (1) Incomplete feature extraction: Pure convolutional neural networks (CNNs) lack global contextual information, while pure Transformers lack local details. DEENet addresses this issue through its convolutional neural network-Transformer dual encoder backbone network. (2) Noise interference in small targets: Single-scale fusion often fails in cluttered backgrounds. We propose a dual-channel fusion (DCF) module to cross-enhance local and global cues, specifically designed to suppress industrial noise. (3) Blurred edge localization: Standard pooling and convolution operations often weaken gradient information. The designed C2f_EEM module introduces a differential operation to explicitly enhance boundary cues.

### Transformer-based and DETR-style detectors

Besides CNN-based methods, Vision Transformer (ViT) models have demonstrated strong capability in modeling long-range dependencies, which is critical for recognizing large-scale defects and those with irregular geometries. Wang et al.^31^ introduced a Swin Transformer module into the one-stage YOLOv5 detection framework to enhance global representation while maintaining real-time efficiency and detection accuracy; however, the robustness of the resulting defect detector still requires further verification under diverse industrial conditions. Liu et al.^32^ proposed a dual-branch network in which a channel-wise global Transformer is employed to model long-range dependencies while preserving local-detail features, yet the overall architecture is relatively complex and its inference efficiency may become a practical bottleneck in deployment. Lv et al.^33^ integrated MobileViTv2 into the YOLOv8 framework to strengthen feature extraction for defects with complex morphology, improving global feature representation while controlling computational cost. Vasan et al.^34^ validated the feasibility of Vision Transformers for steel surface defect classification and further improved performance through hyperparameter configuration and optimization.

In addition, the DETR family, particularly real-time detectors such as RT-DETR^35–37^, simplifies the detection pipeline by removing manually designed components such as non-maximum suppression. Nevertheless, although Transformer-based models are effective at capturing global semantic information, they often under-emphasize fine-grained local textures and edge details, which are critical for characterizing subtle surface defects in steel. This limitation motivates the design of DEENet, which adopts a hybrid architecture to jointly exploit the local precision of CNNs and the global contextual modeling capability of Transformers.

## The overview of methods

**Fig. 2:**
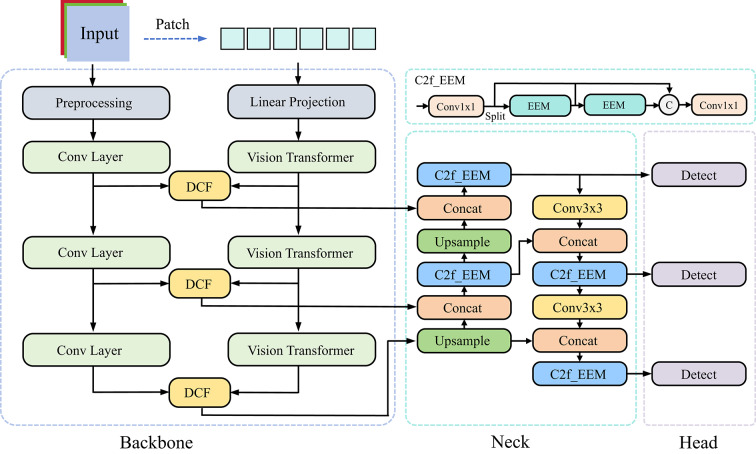
DEENet Network Architecture. The model consists of a dual-encoder backbone (CNN and Transformer branches), a cross-branch Dual Channel Fusion (DCF) module, and an edge-enhanced neck (C2f_EEM). Arrows indicate the data flow, where features from matched stages are fused to combine local textures and global semantics. The $C$ circle denotes the concatenation operation.

YOLOv8 is widely used in industrial inspection for its single-stage efficiency, mature training and inference pipeline, and ease of deployment, making it a practical scaffold for real-time steel surface analysis. Nevertheless, on steel surfaces with diverse and fine-grained defects under cluttered backgrounds, the vanilla pipeline shows limitations in multi-scale feature capture, sensitivity to small targets, and preservation of edge contours. Accordingly, we adopt YOLOv8 as the host framework because it employs a modular C2f-based design while other YOLO versions use different backbones and necks, and we introduce DEENet to address these gaps: a CNN–Transformer dual-encoder extracts complementary local and global representations; a Dual Channel Fusion (DCF) module performs cross-branch fusion to suppress noise and enhance small-defect cues; and an edge-enhancement neck (C2f_EEM) sharpens boundary information with low overhead. The architecture is shown in Fig. 2.

First, the input image is processed by the dual encoder. The convolutional branch captures local textures and fine-grained details, whereas the ViT branch partitions the image into 4 × 4 patches and models long-range dependencies to derive global semantic representations. Second, at matched stages, features from the CNN and ViT branches are fused by the Dual Channel Fusion (DCF) module to suppress irrelevant noise and strengthen small-defect cues under cluttered industrial backgrounds. Finally, we replace the backbone blocks within the C2f units of the neck with the proposed Edge-Enhancement Module (EEM), yielding C2f_EEM. This substitution increases sensitivity to boundary cues while reducing computational overhead, improving localization in edge-blurred scenarios and lowering both parameter count and inference time, thereby providing an accurate and deployable solution for industrial steel surface defect detection. Table 2 shows the tensor data flow of the DEE network at different stages.

**Table 2:** Tensor data streams at different stages of the DEENet.

	Input	Output
Preprocessing / Patch Embedding	(B, 3, 640, 640)	CNN: (B, 64, 320, 320) ViT: (B, 128, 160, 160)
Backbone Stage 1	Previous stage output	CNN: (B, 128, 80, 80) ViT: (B, 256, 80, 80)
DCF Fusion 1	CNN: (B, 128, 80, 80) ViT: (B, 256, 80, 80)	(B, 128, 80, 80)
Backbone Stage 2	CNN: (B, 128, 80, 80) ViT: (B, 256, 80, 80)	CNN: (B, 256, 40, 40) ViT: (B, 512, 40, 40)
DCF Fusion 2	CNN: (B, 256, 40, 40) ViT: (B, 512, 40, 40)	(B, 256, 40, 40)
Backbone Stage 3	CNN: (B, 256, 40, 40) ViT: (B, 512, 40, 40)	CNN: (B, 256, 20, 20) ViT: (B, 512, 20, 20)
DCF Fusion 3	CNN: (B, 256, 20, 20) ViT: (B, 512, 20, 20)	(B, 512, 20, 20)
Neck	(B, 128, 80, 80) (B, 256, 40, 40) (B, 512, 20, 20)	(B, 896, 80, 80) (B, 1664, 40, 40) (B, 2176, 20, 20)

### Dual encoder based on CNN and transformer

DEENet adopts a dual-encoder design, in which the CNN branch is implemented with ResNet-style building blocks, while the Transformer branch is realized using a Vision Transformer (ViT). Conventional convolution operations, constrained by fixed kernel shapes and a static parameterization, often struggle to effectively capture the irregular morphology and heterogeneous textures of real-world defects.

**Fig. 3:**
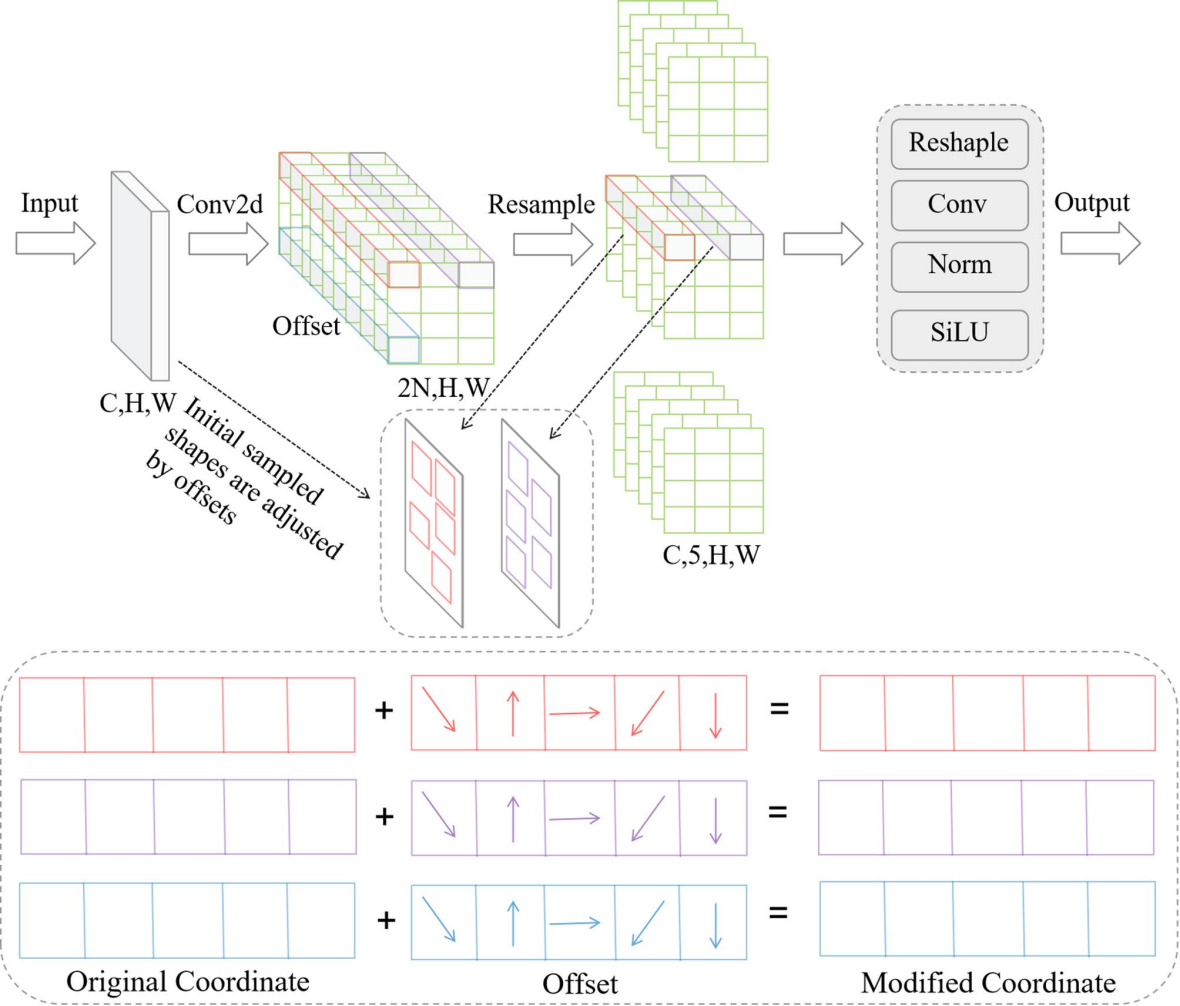
AKConv Network structure.

To mitigate this limitation, we introduce an Adaptive-Kernel Convolution (AKConv)^38^, which leverages flexible sampling patterns and learnable kernel parameterization to better adapt to the characteristics of steel-surface defects. By dynamically aligning the receptive field with local structures, AKConv extracts more discriminative features than fixed-kernel counterparts, thereby strengthening the representational capacity of the CNN branch. The overall architecture of AKConv is illustrated in Fig. 3.

In DEENet, AKConv is integrated into the CNN encoder in the backbone to replace standard convolutions. Unlike traditional convolutions that operate on a fixed $n \times n$ grid, AKConv enables flexible feature sampling. For a given position $p_{0}$ on the output feature map $Y$, its operation can be expressed as follows:

1 $$Y(p_{0} ) = \sum\limits_{n = 1}^{N} {w_{n} \cdot X\left( {p_{0} + p_{n} + \Delta p_{n} } \right)}$$

where $$N$$ represents the number of sampling points, $$w_{n}$$ denotes the learnable weights, $$p_{n}$$ is the predefined grid coordinate, and $$\Delta p_{n}$$ is the learned offset for the $n$-th sampling point.

The fundamental difference between AKConv and Deformable Convolution (DCN) lies in the kernel flexibility. While DCN typically adjusts a standard square grid, AKConv utilizes a coordinate generation algorithm to support an arbitrary number of parameters and arbitrary initial sampling shapes. This allows DEENet to adapt more precisely to the diverse and irregular morphologies of steel surface defects, such as elongated scratches or fragmented inclusions, without being constrained by fixed-size square receptive fields.

### Dual channel fusion module

**Fig. 4:**
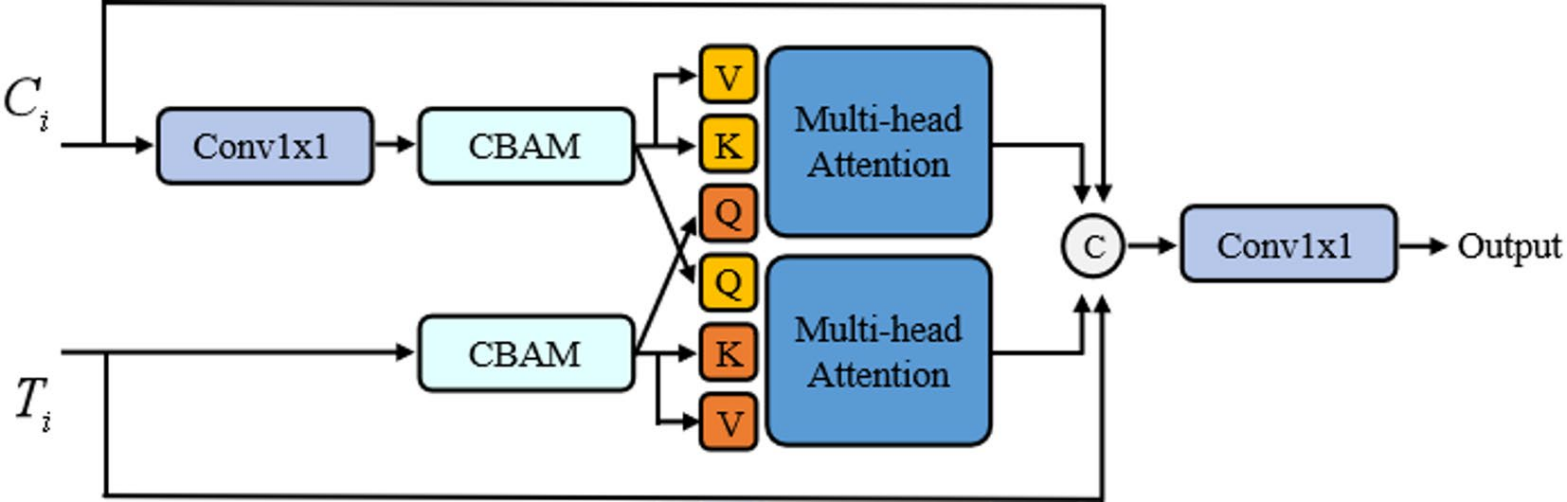
Dual Channel Fusion Module Architecture. $C_{i}$ and $T_{i}$ represent input features from the CNN and Transformer encoders, respectively. $Q$, $K$, and $V$ denote Query, Key, and Value vectors used in the multi-head cross-attention mechanism. The CBAM block performs sequential channel and spatial attention reweighting. The circle with ‘C’ signifies channel-wise concatenation.

As shown in Fig. 4, we propose a Dual-Channel Fusion (DCF) module to integrate the features extracted by the CNN and Transformer branches, thereby achieving complementary enhancement between local details and global semantics. Tailored to the small-object challenge in steel surface defect detection, DCF employs a dynamic feature-integration mechanism that strengthens the model’s sensitivity to minute defects while suppressing interference from cluttered industrial backgrounds. Operationally, features from the two encoders at corresponding hierarchy levels are first aligned in resolution, then undergo cross-branch interaction so that each stream is modulated by cues from the other. The resulting representations are fused with learnable weights and refined through lightweight normalization and residual aggregation, preserving discriminative information without incurring substantial computational overhead. This design enhances small-scale feature expression and improves robustness under complex textures and illumination conditions.

For the CNN-extracted feature ${C}_{i}$, a 1 × 1 convolution is first applied to adjust its channel dimension to match that of the Transformer-extracted feature ${T}_{i}$. This operation standardizes the feature dimensionality, facilitating subsequent fusion and preventing information loss or additional computational overhead due to mismatched dimensions. The specific formulation is:

2 $$C_{i}^{\prime } = {\mathrm{Conv}}_{1x1} \left( {C_{i} } \right)$$

where $\mathrm{Conv}{v}_{1x1}(\cdot )$ denotes the parameterized 1 × 1 convolution kernel, which performs an efficient channel transformation via a learned weight matrix, thereby preserving the CNN’s local texture details. Subsequently, the adjusted CNN feature ${C}_{i}{\prime}$ and the Transformer feature ${T}_{i}$ are respectively fed into the Convolutional Block Attention Module (CBAM), which applies Channel Attention followed by Spatial Attention in a sequential manner to dynamically reweight salient regions of the feature maps. The Channel Attention is computed as:

3 $$M_{c} \left( F \right) = \sigma \left( {{\mathrm{MLP}}\left( {{\mathrm{AvgPool}}\left( F \right) + {\mathrm{MaxPool}}\left( F \right)} \right)} \right)$$

where $\sigma (\cdot )$ denotes the Sigmoid activation function, MLP denotes a multilayer perceptron, and AvgPool and MaxPool extract average- and max-pooled features, respectively; the spatial attention is computed as:

4 $$M_{s} \left( F \right) = \sigma \left( {{\mathrm{Conv}}_{7x7} \left( {\left[ {{\mathrm{AvgPool}}\left( F \right);{\mathrm{MaxPool}}\left( F \right)} \right]} \right)} \right)$$

The CBAM mechanism enhances the saliency of defect-relevant features while suppressing irrelevant noise. In particular, under low-contrast steel-surface conditions, it preferentially highlights the textural differences of small targets, thereby improving the discriminability of the feature representation.

Next, the CBAM-processed features are fed into a multi-head cross-attention mechanism. In the upper branch, the CNN features are used as the Query ($Q$) and Value ($V$), and the Transformer features are used as the Key ($K$); in the lower branch, the roles are reversed to realize bidirectional cross-integration. The specific attention is computed as:

5 $${\mathrm{Attention}}\left( {Q,K,V} \right) = {\mathrm{Softmax}}\left( {\frac{{Q \cdot K^{T} }}{{\sqrt {d_{k} } }}} \right) \cdot V$$

where ${d}_{k}$ denotes the dimensionality of the key. The multi-head mechanism runs multiple attention heads in parallel and concatenates their outputs to further capture diverse dependencies. This cross-attention strategy achieves deep complementarity between local and global features, strengthens the semantic representation of small-scale defects, and mitigates the missed-detection issue of traditional single-encoder models in scenes with densely distributed small targets.

### Edge-enhancement module

Although the C2f module in YOLOv8 exhibits certain advantages for steel surface defect detection—leveraging a Bottleneck structure for efficient feature aggregation and residual connections to preserve multi-level information—it can suffer from missed detections or localization biases when confronting tiny cracks or defects with blurred boundaries, where gradient cues are weak and edge information is easily lost. The conventional C2f module is not well suited to these challenges, leading to a noticeable decline in detection performance.

**Fig. 5:**
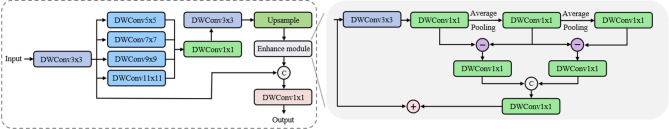
EEM module structure diagram. In the Enhance Module (right), the symbol ‘-’ denotes the element-wise subtraction used to calculate gradient differences between shallow and deep features for boundary highlighting. The symbol ‘ + ’ denotes the residual shortcut addition. DWConv stands for Depthwise Convolution, and AP indicates Average Pooling.

To address this issue, we design an improved C2f module, termed C2f_EEM. Built upon the original C2f, the proposed module replaces the Bottleneck block with our Edge-Enhancement Module (EEM). By introducing a multi-scale feature extraction mechanism, C2f_EEM strengthens the network’s sensitivity to objects of varying sizes. In conjunction with an edge-feature enhancement strategy, it effectively mitigates localization inaccuracies and feature attenuation caused by boundary blur. The structure of the EEM Module is shown in Fig. 5.

In the EEM module, the input first passes through a 3 × 3 DWConv layer for preliminary feature extraction; this depthwise separable convolution reduces computational complexity while capturing local texture details. This design enables efficient extraction of multi-scale preliminary features and avoids the excessive computational cost of standard convolutions. The specific formulation is:

6 $$F_{{{\mathrm{out}}}} = DW{\mathrm{Conv}}_{3x3} \left( {F_{{{\mathrm{in}}}} } \right)$$

Subsequently, the features are distributed in parallel to multiple branches, namely DWConv 5 × 5, DWConv 7 × 7, DWConv 9 × 9 and DWConv 11 × 11. These large-kernel depthwise separable convolutions are designed to capture information under different receptive fields. All output feature maps are then fused via a Concat operation and a DWConv 1 × 1 to integrate features and restore the channel dimension, followed by a DWConv 3 × 3 to produce the final output feature map. Finally, an Upsample operation together with the Enhance Module restores the feature-map resolution and enhances the extraction of edge information.

The Enhance Module comprises two edge-enhancement blocks connected in series and uses differencing to emphasize gradient contrasts between defects and background. The shallow feature ${F}_{a}$ is obtained by applying a 3 × 3 convolution followed by a 1 × 1 convolution to the input feature map $X$ . The deep feature ${F}_{d}$ is produced by applying an average-pooling layer (AP) and a 1 × 1 convolution to the shallow feature ${F}_{a}$. Edge enhancement is then performed separately on the shallow feature ${F}_{a}$ and the deep feature ${F}_{d}$. The resulting feature map $Z$ is differenced with the processed deep feature ${F}_{d}(Z)$, and the output is passed through a 1 × 1 convolution to yield the high-frequency feature map $\phi (Z)$. When $Z$ equals ${F}_{a}$, the shallow edge-enhanced feature $\phi \left({F}_{a}\right)$ is obtained; when $Z$ equals ${F}_{d}$, the deep edge-enhanced feature $\phi \left({F}_{d}\right)$ is obtained. Finally, $\phi \left({F}_{a}\right)$ and $\phi \left({F}_{d}\right)$ are concatenated and fused via a 1 × 1 depthwise convolution, and the result is added element-wise to the input feature map $X$ to produce the final output feature map.

7 $$F_{a} = DW{\mathrm{Conv}}_{1 \times 1} \left( {DW{\mathrm{Conv}}_{3 \times 3} \left( X \right)} \right)$$

8 $$F_{d} = DW{\mathrm{Conv}}_{1 \times 1} \left( {AP\left( {F_{a} } \right)} \right)$$

9 $$\phi \left( Z \right) = DW{\mathrm{Conv}}_{1 \times 1} \left( {Z - F_{d} \left( Z \right)} \right)$$

10 $$Out = X + DW{\mathrm{Conv}}_{1 \times 1} \left( {{\mathrm{Concat}}\left( {\phi \left( {F_{a} } \right),\phi \left( {F_{d} } \right)} \right)} \right)$$

## Experiments

### Dataset and experimental settings

This study uses the NEU-DET dataset^39^ from Northeastern University’s surface-defect database. NEU-DET comprises six typical surface defects observed on hot-rolled steel: rolled-in scale (RS), patches (Pa), crazing (Cr), pitted surface (PS), inclusions (In), and scratches (Sc). Each category contains 300 samples, yielding a total of 1,800 grayscale images with an original resolution of 200 × 200 pixels. For experimental purposes, the dataset was randomly split 8:2 into training and test sets, resulting in 1,440 training images and 360 test images. All images are stored in JPG format. NEU-DET offers sufficient sample diversity and complexity across defect types to enable a comprehensive evaluation of steel surface defect detection algorithms and to support model generalization.

All experiments were conducted on a Windows 10 operating system with an Intel Xeon Gold 5218 CPU, 64 GB RAM, and an NVIDIA GeForce RTX 3090 GPU with 24 GB memory. The implementation was based on the PyTorch 2.0.0 deep learning framework using Python 3.8, and model training was accelerated with NVIDIA CUDA 11.1.

During training, hyperparameters were carefully configured to improve convergence. We adopted the Adam optimizer owing to its adaptive learning-rate properties, with an initial learning rate of 0.003. A learning-rate decay scheme was employed: if the validation loss did not decrease appreciably for ten consecutive epochs, the learning rate was reduced by a factor of 0.1 to facilitate convergence and mitigate entrapment in local minima. The mini-batch size was set to 16, and training proceeded for 1,000 iterations. A weight-decay coefficient of 0.00036 and a momentum parameter of 0.937 were applied. Notably, the confidence threshold for object filtering during inference was set at 0.25, ensuring a robust balance between detection sensitivity and accuracy. The full set of experimental parameters is summarized in Table 3.

**Table 3:** Experimental parameter settings.

Name	Setting
Optimizer	Adam
Initial Learning Rate	0.003
Batch Size	16
Number of Epochs	1000
Decay Rate	0.00036
Momentum Parameter	0.937

### Evaluation metrics

To assess both detection accuracy and speed for strip-steel surface defect detection, we adopt Recall ($R$), Precision ($P$), Average Precision (AP, ${P}_{A}$), and mean Average Precision (mAP, ${P}_{mA}$), with computations given in Eqs. (11), (12), (13) and (14).

11 $$R = \frac{{T_{P} }}{{T_{P} + F_{N} }}$$

12 $$P = \frac{{T_{P} }}{{T_{P} + F_{P} }}$$

13 $$P_{A} = \int\limits_{0}^{1} {P\left( R \right)dR}$$

14 $$P_{mA} = \frac{{\sum\nolimits_{i = 0}^{n} {P_{A} \left( i \right)} }}{n}$$

In these definitions, ${T}_{P}$ denotes the number of true positives, ${F}_{N}$ the number of false negatives, ${F}_{P}$ the number of false positives, and $n$ the total number of classes. We also report computational cost (GFLOPs), parameter count (Parameters), and inference throughput (Frames Per Second, FPS), which respectively characterize computational efficiency, model capacity, and processing speed.

### Comparison between the proposed method and YOLOv8

To directly evaluate the effectiveness of the proposed approach, we conducted experiments on the NEU-DET dataset and recorded the results of YOLOv8 and DEENet for side-by-side comparison.

As shown in Table 4, the DEENet algorithm achieves a significant improvement in detection accuracy for the various defect categories. Specifically, for Crazing the detection accuracy increases from 43.9 to 53.5%, a gain of 9.6 percentage points. For Inclusion and Patches, the accuracies rise from 83.6 to 88.3% and from 92.6% to 96.8%, with increases of 4.7 and 4.2 percentage points. In the remaining categories of Pitted Surface, Rolled-in Scale and Scratches, the accuracies improve from 82.8 to 87.3%, 66.3 to 68.8% and 89.9 to 93.7%, corresponding to gains of 4.5, 2.5 and 3.8 percentage points. Based on the above experimental results, the Dual Channel Fusion module, through complementary fusion of local and global features, addresses the problem of a high miss-detection rate for Crazing caused by the small target size and dense distribution. The advantage of the dual-encoder structure in handling irregularly shaped defects is reflected in the Inclusion category. C2f_EEM, by using multi-scale depthwise separable convolutions and a large-kernel design together with the differencing operation in the Enhance Module, highlights high-frequency edge features, avoids edge weakening, and improves the detection accuracy for Patches, Pitted Surface, Scratches, and Rolled-in Scale defects.

**Table 4:** Comparison of detection results of YOLOv8 and DEENet for each defect type.

Methods	Crazing	Inclusion	Patches	Pitted Surface	Rolled-in Scale	Scratches
YOLOv8	43.9	83.6	92.6	82.8	66.3	89.9
DEENet	**53.5**	**88.3**	**96.8**	**87.3**	**68.8**	**93.7**

Significant values are in bold.

**Fig. 6:**
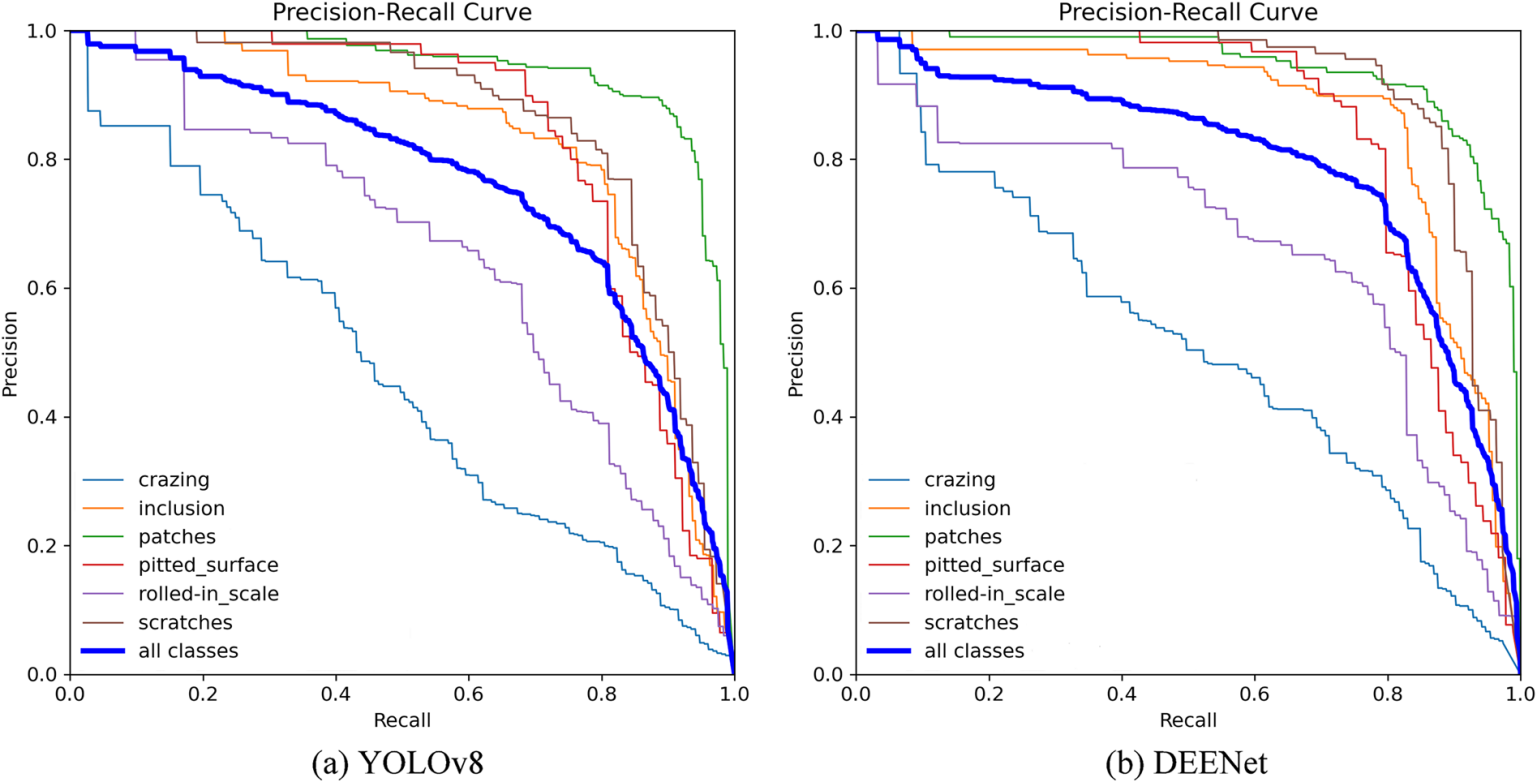
P-R curves before and after improvement of YOLOv8 algorithm.

As shown in Fig. 6, the precision–recall (P–R) curves for YOLOv8 and DEENet further substantiate these improvements. In the high-recall regime, DEENet’s precision curve consistently lies above that of YOLOv8, indicating a substantial reduction in false positives while maintaining comprehensive recall. The advantage is especially pronounced when recall exceeds 0.8, demonstrating the practicality and reliability of DEENet for industrial applications.

**Fig. 7:**
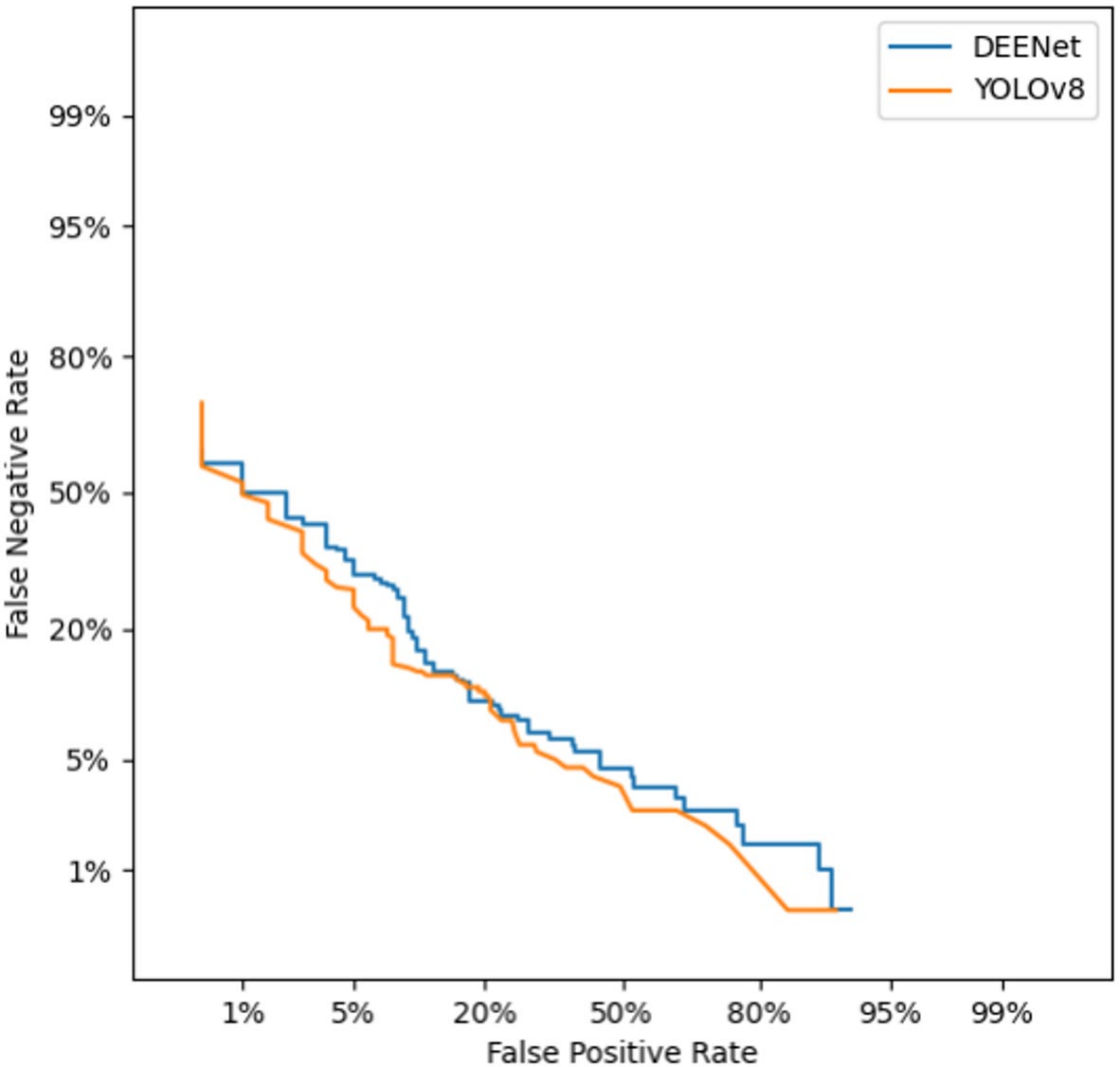
Comparison of DET curves between Yolov8 and DEENet.

To further evaluate the reliability of the model in industrial applications, Fig. 7 shows a comparison of the detection error trade-off (DET) curves between the YOLOv8 baseline model and DEENet. Compared to YOLOv8, DEENet’s curve is closer to the lower left corner of the coordinate axis, indicating that at the same false positive rate level, DEENet can maintain a lower false negative rate, achieving a better error trade-off. This is mainly due to the effective modeling of global semantics by the dual-encoder and the enhancement of edge information by the C2f_EEM module, enabling the model to extract identifiable features even when facing defects with extremely low contrast or small shapes (such as Crazing), significantly reducing the risk of false negatives. Experimental results demonstrate that DEENet has higher detection stability in complex industrial environments. Through the cross-enhancement of local and global features by the Dual Channel Fusion (DCF) module, DEENet effectively suppresses background noise interference, providing a more accurate and reliable visual judgment basis for automated production lines.

### Model performance comparison

To evaluate the performance of different models for steel surface defect detection, we experimented with several representative algorithms; the comparative results are summarized in Table 5. Relative to the two-stage Faster R-CNN and the single-stage SSD, DEENet improves mAP by 20.8% and 9.0%, and increases recall by 9.3% and 0.8%, respectively. While maintaining only 8.2M parameters and 12.4G FLOPs, DEENet also surpasses the YOLO family baselines and RT-DETR in both mAP and recall. Under identical hyperparameter settings, our method yields mAP gains of 11.1% over YOLOv5s, 7.7% over YOLOv9, 9.6% over YOLOv10, and 7.6% over YOLOv11. Moreover, despite its low computational cost, DEENet achieves notably higher precision and F1 score than the YOLOv10 baseline. DEENet also exhibits markedly lower computational complexity than RT-DETR, with FLOPs as low as 12.4G.

**Table 5:** Comparative experimental results of models on the NEU-DET dataset.

Methods	mAP/%	Precision/%	Recall/%	F1-score/%	Param/M	FLOPs/G
Faster RCNN	60. 6	77.9	76.3	78.9	60. 1	246.4
SSD	72. 4	79.3	84.8	82.0	25. 0	64.2
YOLOv5s	70. 3	78.5	78.5	81.2	**7. 2**	27.7
YOLOv9	73. 7	79.6	79.9	80.8	12. 1	32.9
YOLOv10	71. 8	80.3	81.4	81.3	8. 0	40.6
YOLOv11	73. 8	79.9	79.8	80.0	9. 4	42.8
RT-DETR^40^	75.0	79.3	81.4	79.9	42	136
MSD-YOLO^41^	80.9	83.2	82.4	84.9	35.3	54.2
MD-YOLO^42^	78.2	82.6	81.6	82.1	9.0	14.1
DEENet	**81.4**	**84.8**	**85.6**	**85.2**	8.2	**12.4**

Significant values are in bold.

As shown in Table 5, on the NEU-DET dataset the DEENet model performs well on multiple defect categories, especially achieving detection accuracies of 56.5% and 96.8% on the Cr and Pa categories, which are significantly higher than the 37.9% and 91.5% of Faster R-CNN, the 38.7% and 88.5% of SSD, and the corresponding values of the YOLO-series baseline models. Compared with YOLOv5s, DEENet improves the Cr, In, Pa, Ps and Sc categories by 10.5, 6.3, 5.8, 3.3 and 3.9%, respectively; compared with YOLOv10, its increases on Cr, In, Pa and Ps reach 7.3, 6.7, 3.4 and 15.2%. Although it is slightly lower than some models on the Rs category, the overall performance is balanced, and it shows advantages especially on small targets and defects with blurred edges (such as Cr and Ps). In addition, compared with advanced models such as MSD-YOLO and MD-YOLO, DEENet also achieves better results on In, Pa, Ps and Sc, with a higher average accuracy. These results show that, by using the dual-encoder structure and the edge-enhancement module, DEENet effectively improves the robustness of multi-scale and small-target detection, and is suitable for high-precision defect recognition in complex industrial scenarios.

As indicated in Table 6, the Crazing (Cr) category consistently exhibits the lowest detection accuracy across all evaluated models. A deeper analysis reveals that Crazing manifests as high-density, fine-grained, and net-like cracks with extremely low contrast against the steel background. Traditional models like Faster R-CNN and early YOLO versions struggle because the standard pooling and stride operations tend to discard these subtle high-frequency details during deep feature extraction. In contrast, DEENet achieves a significant gain of 10.5% over YOLOv5s and 7.3% over YOLOv10 in this category. This improvement is primarily attributed to two factors: (1) the Transformer branch maintains long-range spatial dependencies, preventing the total loss of sparse crack information, and (2) the C2f_EEM module explicitly sharpens the faint gradient differences between the cracks and the normal surface through its difference operation.

**Table 6:** Comparative experimental results of models for each defect type on the NEU-DET dataset.

Methods	Cr	In	Pa	Ps	Rs	Sc
Faster RCNN	37.9	77.8	91.5	80.4	60.2	89.6
SSD	38.7	76.8	88.5	78.0	65.4	77.4
YOLOv5s	46.0	82.0	91.0	84.0	71.4	89.8
YOLOv9	46.2	80.1	95.4	80.0	72.2	91.2
YOLOv10	49.2	81.6	93.4	72.1	68.3	85.3
YOLOv11	44.4	81.7	94.8	82.1	70.5	93.6
RT-DETR^40^	45.5	85.7	91.8	83.7	67.8	91.3
MSD-YOLO^41^	56.3	84.3	92.0	83.1	72.3	97.7
MD-YOLO^42^	46.7	81.4	91.3	85.1	**72.6**	92.0
DEENet	**56.5**	**88.3**	**96.8**	**87.3**	68.8	**93.7**

Significant values are in bold.

### Ablation studies

To verify the effectiveness of the improved YOLOv8 CCD algorithm, five groups of comparative ablation experiments were carried out using YOLOv8 as the baseline model. The experimental results are shown in Table 7, where “√” indicates that the module is used and “–” indicates that the module is not used.

**Table 7:** Comparison of ablation experiment results for different module performance parameters.

Methods	Dual-Branch Backbone	DCF	C2f_EEM	mAP(%)	FLOPs/G	Param/M
1	√	–	–	78.6	8.0	10.3
2	–	–	√	78.5	8.1	11.5
3	√	√	–	80.5	8.2	11.2
4	√	–	√	80.0	8.1	11.5
5	√	√	√	**81.4**	**8.2**	**12.4**

Significant values are in bold.

As shown in Table 7, Method 1 represents the model with the Dual-Branch Backbone module introduced, with an mAP of 78.6%, FLOPs of 8.0G, and Param of 10.3 M. Method 2 uses only the C2f_EEM module, making the mAP reach 78.5%, with FLOPs and Param slightly increased, which proves the effectiveness of the C2f_EEM module in the steel surface defect detection task. Method 3 combines the Dual-Branch Backbone and DCF modules; compared with Method 1, the mAP increases by 1.9%, and FLOPs and Param increase slightly, indicating that the DCF module improves the accuracy and efficiency of defect target detection. Method 4 adopts the Dual-Branch Backbone and C2f_EEM modules, achieving an mAP of 80.0%, with FLOPs and Param basically stable, which shows that this combination has a certain effectiveness in improving detection accuracy. Method 5, namely the proposed DEENet algorithm, combines the Dual-Branch Backbone, DCF, and C2f_EEM modules, increasing the mAP from 76.6% of the baseline model to 81.4%, an increase of 4.8%, with FLOPs of 8.2G, although Param increases slightly. Overall, compared with the original YOLOv8 algorithm, the proposed DEENet algorithm for steel surface defect detection maintains high detection accuracy while reducing computational load, and has practical value in real-world steel surface defect detection applications.

### Comparative study of activation functions

**Fig. 8:**
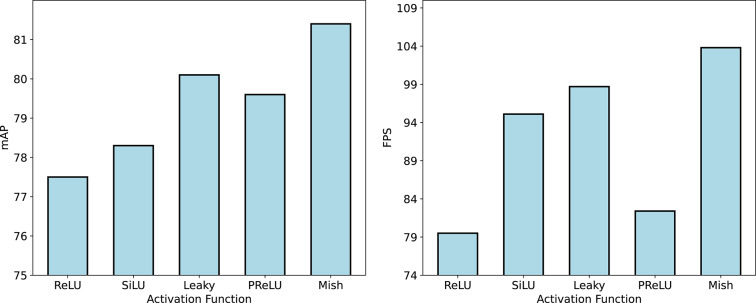
Comparative experimental results of different activation functions.

To investigate the impact of different activation functions on the performance of DEENet, five activations, namely ReLU, SiLU, Leaky ReLU, PReLU, and Mish, were compared. For experimental purposes, the Mish activation in DEENet was replaced by ReLU, SiLU, Leaky ReLU, and PReLU. The comparative results are shown in Fig. 8.

In Fig. 8, the computational complexity and parameter counts remain identical across all five activations, indicating that changing the activation does not materially affect these measures. Using ReLU yields an mAP of 77% and FPS of 79. SiLU and Leaky ReLU provide moderate improvements over ReLU, reaching 78% and 79% mAP with 94 and 84 FPS, respectively. PReLU outperforms SiLU, ReLU, and Leaky ReLU, increasing mAP by 2, 3, and 1 percentage points, with FPS of 99. Compared with the other four activations, Mish delivers the best overall performance, achieving an mAP of 81% and the highest FPS of 104. Overall, Mish yields the highest mAP and FPS among the tested activations, providing the best trade-off between detection accuracy and inference speed without increasing model complexity. This advantage makes DEENet more suitable for accurate and efficient steel surface defect detection in practical industrial scenarios, so Mish is adopted as the activation function in the final model.

### Further analysis

To visually assess DEENet’s performance gains and convergence behavior relative to YOLOv10 during training, we compare the evolution of precision, recall, mAP, and loss curves, thereby verifying the effectiveness of the proposed optimizations.

**Fig. 9:**
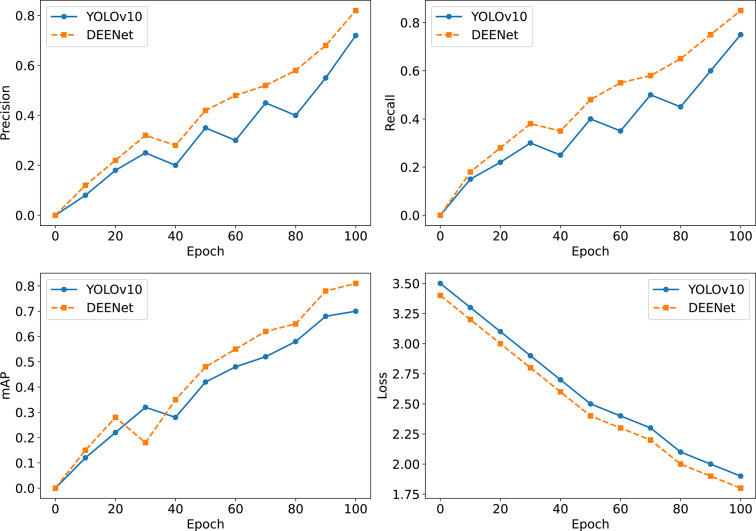
Comparison of the training process between YOLOv10 and DEENet.

As shown in the loss curves in Fig. 9, DEENet exhibits a larger decrease and reaches a stable state faster than YOLOv10. Although this figure focuses on the first 100 epochs to highlight optimization efficiency, according to Table 3, the model was trained for a total of 1000 epochs, with the loss plateauing after the initial period, ensuring that performance reached its potential limits. In the precision curve, the precision of YOLOv10 increases slowly from about 0.0 at the beginning, with obvious fluctuations; in contrast, the precision curve of DEENet starts at a similar level but rises faster and with smaller fluctuations, indicating that the improved modules enhance the stability of feature extraction and improve the model’s ability to accurately recognize defects. The recall curves show a similar trend: the recall of YOLOv10 increases gradually, while DEENet shows a smoother increase, especially around epochs 40–60, reflecting that the dual-channel fusion improves the recall of small and multi-scale defects. The mAP curves further confirm this advantage and highlight the role of the edge-enhancement module in improving the overall detection accuracy. The loss curves show that both models decrease from their initial values, but DEENet drops faster, indicating that the improved model converges more quickly and that the training process is more efficient.

**Fig. 10:**
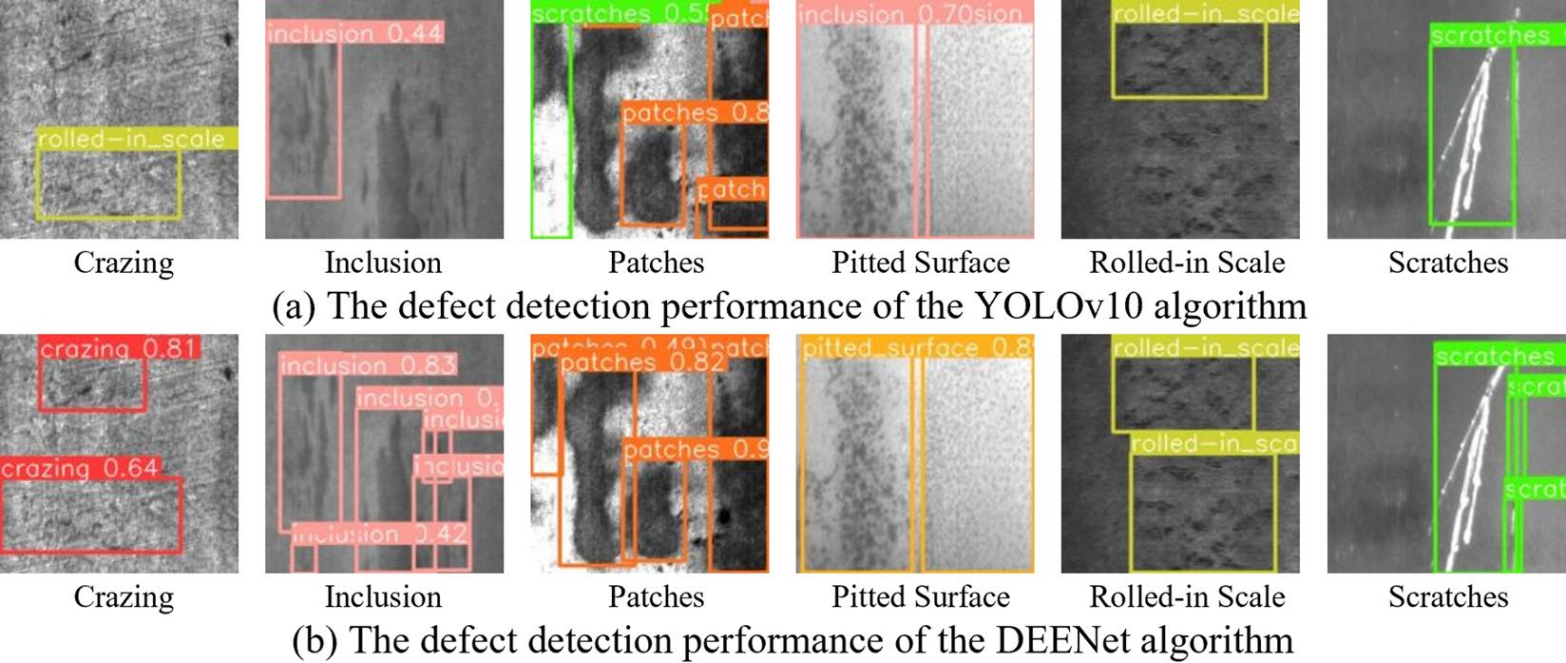
Defect detection performance of YOLOv10 algorithm before and after improvement.

To enable a direct comparison between DEENet and YOLOv10, we randomly sampled images from the dataset for defect detection; representative results are shown in Fig. 10.

For Crazing, YOLOv10 produces loose detection regions that include extraneous background, whereas DEENet yields tighter boundaries with fewer false positives, highlighting the edge-enhancement module’s precise capture of fine cracks. For Inclusion, YOLOv10 exhibits overlapping boxes and low confidence, while DEENet reflects the complementary effect of dual-channel fusion on multi-scale features. Under Patches, YOLOv10 shows blurred boundaries, whereas DEENet provides clearer delineation. Similar trends are observed for Pitted Surface, Rolled-in Scale, and Scratches. Overall, across all six defect categories, DEENet achieves higher accuracy than YOLOv10, with improvements in confidence, boundary precision, and defect coverage. YOLOv10 often produces loose or overlapping bounding boxes that fail to encompass the entire crack region, whereas DEENet generates much tighter and more accurate boundaries. This visual evidence substantiates that the proposed edge-enhancement module effectively captures the fine crack structures that are otherwise missed by conventional backbones. These results substantiate the effectiveness of the proposed modules for complex steel-surface defects and provide more reliable visual evidence for industrial inspection.

### Generalization analysis on GC10-DET dataset

To further verify the generalization capability of DEENet, we conducted experiments on the GC10-DET dataset, which features diverse industrial surface defects. As shown in Table 8, DEENet maintains a competitive mAP of 71.5%, outperforming YOLOv10 by 1.1%. This consistency across different datasets demonstrates that our dual-encoder architecture and edge-enhancement strategy are not limited to NEU-DET but are robustly applicable to varied industrial inspection tasks.

**Table 8:** Comparative experimental results of models for each defect type on the GC10-DET dataset.

Methods	mAP/%	Precision/%	Recall/%	Param/M	FLOPs/G
YOLOv5s	64. 3	68.1	53.2	**7. 2**	27.7
YOLOv9	68. 7	64.3	55.7	12. 1	32.9
YOLOv8	69.2	65.7	63.5	12.4	**8.2**
YOLOv10	70. 4	69.7	70.1	8. 0	40.6
YOLOv11	69. 7	69.2	68.2	9. 4	42.8
RT-DETR^33^	69.4	68.8	68.6	42	136
MSD-YOLO^34^	65.6	63.9	64.3	35.3	54.2
MD-YOLO^35^	69.3	69.0	70.4	9.0	14.1
DEENet	**71.5**	**70.3**	**71.6**	8.2	12.4

Significant values are in bold.

### Robustness analysis

**Fig. 11:**
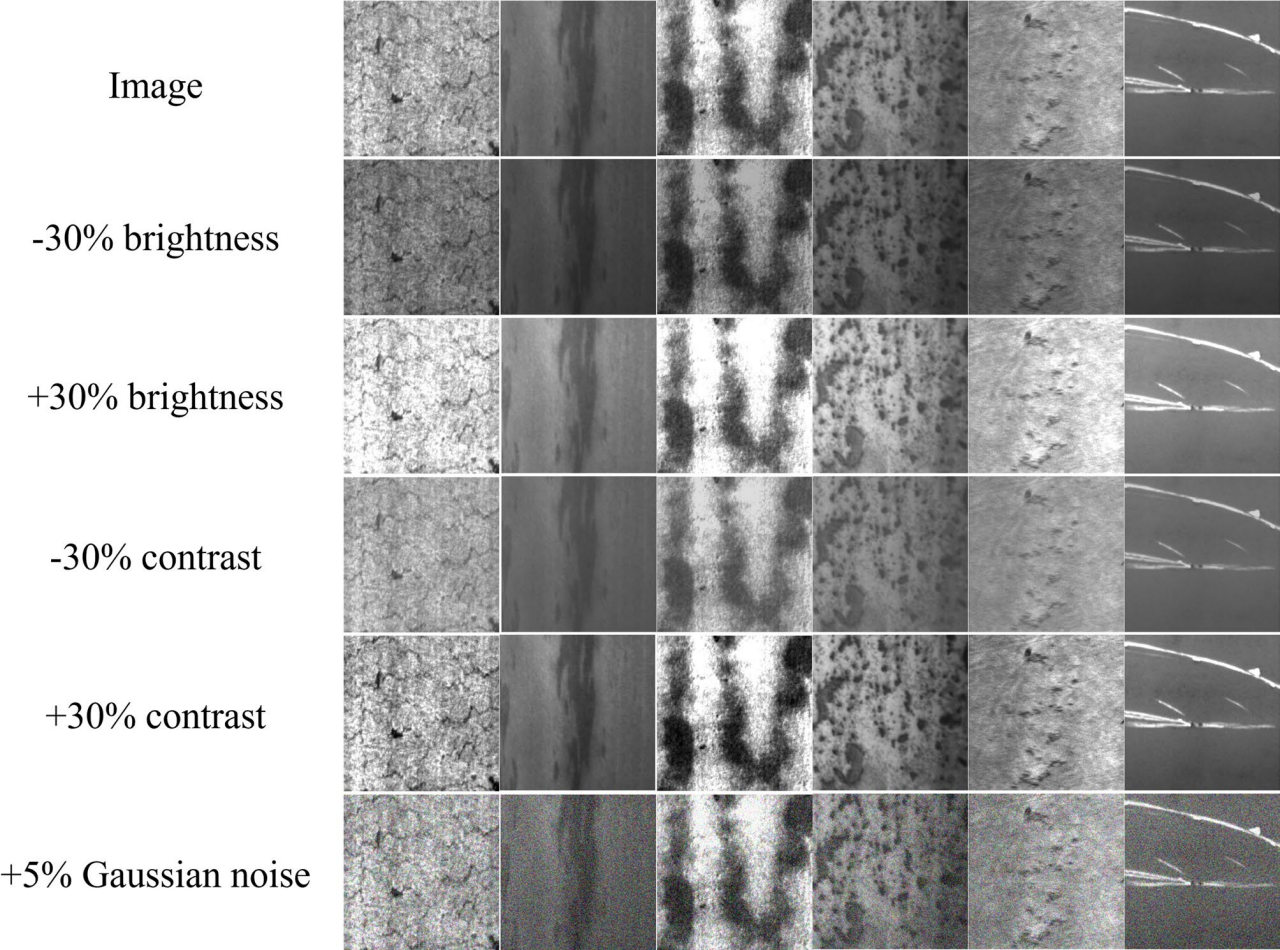
Comparison of surface defect images of strip steel after adding perturbation.

We simulated interference in a real industrial environment by applying Gaussian noise to the test set and adjusting the brightness. In the disturbance type, we set the brightness to decrease by 30%, increase by 30%, decrease the contrast by 30%, increase the contrast by 30%, and add 5% Gaussian noise. Figure 11 shows the surface defect image of the strip after adding perturbation.

As shown in Table 9, even in extreme cases where low brightness (-30% brightness) causes texture blurring or Gaussian noise (5% Gaussian noise) leads to detail loss, DEENet still effectively captures key defect features. This excellent robustness is mainly attributed to the model’s dual-branch architecture: the Transformer encoder ensures semantic stability under global disturbances by modeling long-range dependencies, while the C2f_EEM module enhances the gradient information of defect edges through differentiation operations, thus achieving accurate boundary localization in low-contrast backgrounds. When various environmental disturbances are introduced, although the various indicators fluctuate, the overall decline is limited to a low range. DEENet not only performs excellently on standard datasets but also possesses generalization value and practicality when facing diverse industrial production environments.

**Table 9:** NEU-DET dataset detection results after image perturbation.

	Precision	Recall	mAP
Image	84.8	85.6	81.4
− 30% brightness	79.7	71.3	78.6
+ 30% brightness	84.6	81.4	80.5
− 30% contrast	81.0	79.3	79.1
+ 30% contrast	81.9	78.8	79.7
− 5% Gaussian noise	80.4	77.6	78.4

## Discussion

The superior performance of DEENet is primarily attributed to the synergy between its core modules. The dual-encoder backbone successfully captures both local textures and global context, while the DCF module integrates these features to suppress industrial noise. Furthermore, the C2f_EEM module’s ability to sharpen boundary information through differential operations significantly mitigates localization inaccuracies in edge-blurred scenarios.

However, this study has several limitations. First, the parameter count remains relatively high, which could be a bottleneck for deployment on resource-constrained edge devices. Second, the current evaluation is primarily based on the NEU-DET dataset, lacking validation against the diverse noise found in broader real-world industrial environments. Lastly, the model’s robustness under extreme illumination or occlusion still requires optimization.

Future work will focus on model lightweighting through techniques such as knowledge distillation or pruning. We also plan to explore multimodal fusion—such as integrating infrared imagery—to enhance the model’s generalization across various industrial settings. Furthermore, incorporating adaptive learning strategies will be essential for improving responsiveness to dynamic defect scenarios.

## Conclusion

This research introduced DEENet, a novel dual-encoder model designed to address the challenges of insufficient feature extraction, weak small-object detection, and blurred edge perception in steel surface inspection. By integrating a CNN-Transformer backbone, a Dual Channel Fusion (DCF) module, and the C2f_EEM edge-enhancement module, DEENet achieves high-precision detection in complex industrial scenarios. Experimental results on the NEU-DET dataset demonstrate that DEENet achieves a superior mean average precision (mAP) of 81.4%, significantly outperforming existing baseline models in terms of accuracy, recall, and convergence speed. These findings validate the effectiveness of the proposed approach and provide a valuable reference for advanced defect inspection in modern steel production lines.

## Data Availability

The publicly available dataset utilized in this research can be accessed via the following link: https://www.kaggle.com/datasets/kaustubhdikshit/neu-surface-defect-database.
